# Impact of Cellulolytic Fungi on Biodegradation of Hemp Shives and Corn Starch-Based Composites with Different Flame-Retardants

**DOI:** 10.3390/microorganisms10091830

**Published:** 2022-09-14

**Authors:** Dovilė Vasiliauskienė, Renata Boris, Giedrius Balčiūnas, Agnė Kairytė, Jaunius Urbonavičius

**Affiliations:** 1 Department of Chemistry and Bioengineering, Vilnius Gediminas Technical University, LT-10223 Vilnius, Lithuania; 2 Laboratory of Composite Materials, Institute of Building Materials, Faculty of Civil Engineering, Vilnius Gediminas Technical University, LT-08217 Vilnius, Lithuania; 3 Laboratory of Thermal Insulating Materials and Acoustics, Institute of Building Materials, Faculty of Civil Engineering, Vilnius Gediminas Technical University, LT-08217 Vilnius, Lithuania

**Keywords:** biocomposite, hemp shives, corn starch, *Rhizopus oryzae*, *Aspergillus fumigatus*, cellulolytic fungi, mechanical performance

## Abstract

Biocomposite boards (BcBs) composed of hemp shives and corn starch are known as thermal insulating or structural building materials. Therefore, they must be stable during exploitation. However, BcBs are exposed to microorganisms present in the environment, and it is of great interest to investigate the biodegradation behaviour of these materials. This work identified microorganisms growing on BcBs that contain either Flovan CGN or expandable graphite as flame retardants and selected fungi such as *Rhizopus oryzae* and *Aspergillus fumigatus* to test the way they affect the materials of interest. For this purpose, the enzymatic activity of cellulases and amylases produced by these organisms were determined. In addition, the apparent density as well as compressive strength of the affected boards were evaluated. The results showed that apparent density and compressive strength deteriorated in BcB composition with the Flovan CGN flame retardant. At the same time, the level of deterioration was lower when the expandable graphite was used, suggesting that it also acts as an antimicrobial agent. A scanning electronic microscopy analysis was employed to monitor the growth of microorganisms in the BcBs. Such analysis demonstrated that, regardless of BcB composition, fungi easily penetrate into the middle layers of the material.

## 1. Introduction

Sustainable development and bioeconomy are becoming apparent and prominent. Therefore, biocomposite boards (BcBs) have a niche in the building materials sector with a potential for further growth, and many studies have been devoted to development and analysis. These BcBs are advantageous thanks to the natural properties of natural fibres and particles [1]. Additionally, the reusing and recycling of biocomposite materials are largely acknowledged by both industrial and scientific communities with regard to the depletion of non-renewable feedstock. From this point of view, BcBs produced from natural fibres and biopolymers are of great importance due to their ability to attain the required properties and functionalities necessary for the final product at a reasonable cost [2]. Such BcBs and their bio-based components can be easily recycled, reused or disposed at the end of their service life without contaminating the environment, which would be impossible if composites were produced using synthetic biodegradable materials.

Naturally occurring plant particles are the most common materials chosen as aggregates for the production of BcBs. The main plants used in the production of BcBs are flax, jute, hemp and kenaf [3,4,5,6]. Hemp is the second-largest source of fibres after jute, and it has gained substantial attention as a reinforcement and aggregate in biopolymer matrices during the last decade because of its renewability and recycling properties [7]. These materials also biodegrade easily compared to materials produced using non-renewable feedstock [8].

The microbiological susceptibility of BcBs is dependent on their pH. It was shown [9] that BcBs with a lime-based binder material had no fungi or bacteria growth due to the alkaline nature of raw materials used for the production of BcBs. When the pH level of the raw materials decreases, the microorganisms grow better. Moreover, BcBs are produced not only with inorganic binding materials; they also include starch- or protein-based polymers, polylactic acid, polyhydroxyalcanoates, polycaprolactone, etc. [10]. Among all biodegradable polymers, the most abundant is starch [11,12]. It is an inexpensive, renewable carbohydrate polymer, which showed promising results for application in the building materials sector [13].

Hemp shives (HS), as an aggregate, and starch, as a binding material for BcBs production, were studied by several research groups [14,15,16], who have developed BcBs with light, porous and solid structures with a sufficient mechanical performance, low thermal conductivity and high flame retardancy for application as a building material for thermal insulation. However, such BcBs are susceptible to water or water vapours as well as biodegradation. Therefore, it is necessary to assess how the performance characteristics of such BcBs change after exposure to microorganisms. In addition, high humidity can cause “sick building syndrome” (SBS), whereby microorganisms can cause adverse health effects such as allergies, infections and toxicity that appear to be linked with the time spent in a building [17,18]. It is known that HS are very hydrophilic because the hydroxyl groups are responsible for water sorption. The hemp cell wall is rich in amorphous polysaccharides such as hemicelluloses (~15%) and pectin (~6%) [19]. Therefore, BcBs have many nutrients for microorganisms that are very important for the decomposition of plant materials in nature. The spores or fungi of these microorganisms may already be present in building materials (walls, partitions, ceilings). Furthermore, they can enter the house via the ventilation systems. Their growth is determined by various factors, but most commonly by flooding (from the roof or the pipes), and also in the case of poor ventilation in the premises (kitchen, bathroom). With excess water and enough oxygen available, microorganisms multiply very quickly, dispersing spores and mycelium [20]. Their growth rate depends on the species, water content and activity and relative humidity. Microbial growth can make BcBs an unattractive alternative to traditional building materials. It is important to know how long BcBs can be in service, because fungi are the perfect decomposers of lignocellulosic materials in nature [21].

The current study investigates HS and corn starch (CS)-based BcBs with two different flame retardants—phosphorus and nitrogen containing Flovan CGN, as well as expandable graphite. Some surface treatments applied or additives used to improve the performance (flame-retardants, water repellents, etc.) can lead to the inhibition of anaerobic digestion [22]. Therefore, the impact of two different flame retardants on the biodegradability of BcBs as well as their mechanical performance before and after the impact of microorganisms were determined and evaluated.

## 2. Materials and Methods

### 2.1. Raw Materials

In order to produce BcBs, HS with a fraction of 2.5–5 mm obtained from the hemp fibre separation process (Rokiškis district, Lithuania) were used as an aggregate, and CS having a bulk density of 550 kg/m^3^ (Roquette, Lestrem, France) was incorporated as a binding material. Expandable graphite 9950250 nitric acid (G) flakes (ProGraphite GmbH, Untergriesbach, Germany) with an average particle size min. 70% of 300 µm and Flovan CGN-01 (F) (Huntsmann, Basel, Switzerland) were incorporated as solid and liquid flame retardants. Boiling water was used to activate thin fibres on HS surface, increase and strengthen the interfacial adhesion between aggregate and binding materials [16].

### 2.2. Preparation of BcBs

BcBs were obtained according to the composition presented in Table 1 by pressing the raw materials mixture to 40 vol.% at 0.8 MPa pressure.

Metal screws were used to support the loaded samples in the mould, which was then put into a ventilated oven for the three-stage thermal treatment process: first stage—within 1 h, the temperature was raised to 160 °C, second stage—160 °C temperature was maintained for 6 h and third stage—temperature was decreased. After the thermal treatment, the mould with the hardened product was left in the thermal treatment chamber until the temperature reached the conditions of the surrounding environment. One batch of BcBs was prepared with G (G composition), one batch with F (F composition) and one batch without additives (control composition).

### 2.3. Isolation and Identification of Bacterial and Fungal Strains

The microbial strains were isolated from 5-year-old biocomposite board made of hemp shives (2–5 mm) and acrylic resins as the binder (the piece of board of 2.5 × 2.5 × 1 cm was used). The board was stored at natural room temperature and humidity conditions. The board was moistened with 1 mL of sterile water over the whole surface and left at room temperature (22 °C) for 7–21 days. After that, the microorganisms were isolated on agar plates as pure cultures and stored in 20% glycerol at −80°C temperature. In total, five bacterial species and one genus were identified; for fungi, three isolates were identified at the level of genus, whereas the remaining 11 were of the species level.

To evaluate the morphology of *Pseudomonas putida*, they were imaged after dilution in 0.9% NaCl solution and stained using a Gram-staining kit (Thermo Scientific Remel, Lenexa, KS, USA). The images were taken using the Olympus CX 41 (Olympus Europa SE & Co., Hamburg, Germany) microscope and processed using PixeLINK µScope Profesional (Ottawa, ON, Canada) software. *Aspergillus fumigatus* culture was stained with the lactophenol blue solution (Merck, Darmstad, Germany). The images were taken using an Olympus CX 41 (Olympus Europa SE & Co., Hamburg, Germany) microscope and processed using PixeLINK µScope Profesional (Ottawa, ON, Canada) software (2013 Professional version). *Rhizopus oryzae* were grown on the BcBs. Then, the culture was diluted with water, and images were taken using Levenhuk DTX 90 (China) and processed using ToupView software (Kelvin Grove, Australia, free access).

For identification of the bacteria, a colony of pure culture growing on LB-agar medium was mixed with 20 µL of nuclease-free water in a 1.5 mL Eppendorf-type tube. One µL of this suspension was used in a PCR mixture with DreamTaq DNA polymerase (Thermo Fisher Scientific Baltics, Vilnius, Lithuania) according to the manufacturer’s instructions with universal primers according to the V1-V9 region 16S rRNA 27F (5′-AGAGTTTGATCMTGGCTC-3′) and 1492R (5′-TACGGYTACCTTGTTACGACTT-3′).

In order to identify the isolated fungal strains, rDNA internal transcribed spacers (ITS) were amplified using the specific primers ITS1 forward (TCCGTAGGTGAACCTGCGG) and ITS4 reverse (TCCTCCGCTTATTGATATGC). Pure colonies growing on PDA agar media after 7–21 days incubation were obtained with a sterile disposable microbiological loop of spores or mycelium and mixed with 100 µL of non-nuclease water in a 1.5 mL Eppendorf-type tube, then heated for 5 min. at 95 °C; 1 µL of this suspension was added into the PCR reaction mixture with DreamTaq DNA polymerase (Thermo Fisher Scientific Baltics, Vilnius, Lithuania).

The quality of PCR products was determined using 1% agarose gel electrophoresis, with the size of the products being about 600–800 kb for fungi and 1500 kb for bacteria. The PCR products were purified using a GeneJet PCR purification kit (Thermo Fisher Scientific Baltics, Vilnius, Lithuania).

The purified PCR products were sequenced at Macrogen (Amsterdam, The Netherlands). The obtained sequences were compared with those found at the National Center for Biotechnology Information (NCBI) using the BLAST sequence analysis tool. After removing the vector sequences, they were submitted to GenBank. The accession numbers were obtained and are provided only for those microorganisms that were used for further experiments. These numbers are: for *P. putida*—OP247628; *A. fumigatus*—OP247619; *Rh. oryzae*—OP271776.

### 2.4. Preparation of Microorganisms for Incubation on the BcBs

Bacterium *Pseudomonas putida* were incubated in liquid LB medium (15 g/L agar (Formedium, Swaffham, UK), 10 g/L NaCl (Carl Roth, Karlsruhe, Germany), 5 g/L yeast extract (Sigma-Aldrich, Poznan, Poland) at 30 °C temperature for 24 h, and then the suspension of 10^9^ colony forming units (cfu)/mL in 0.9% NaCl were prepared. Fungi *Rhizopus oryzae* were incubated on the PDA (VWR BDH Chemicals, Darmstadt, Germany) media for 10–21 days, and spores were collected in 20 mL of 0.9% NaCl and diluted to 10^6^ units/mL. The *P. putida* and *Rh. oryzae* suspensions were mixed in equal parts, with 1 mL poured onto three Bcb samples, and incubated for 6 months. For the first three weeks, the images were made every week, and afterwards every month.

The apparent density of the BcBs before, during and after incubation was determined in accordance with EN 1602 [23] requirements, and linear dimensions were measured based on EN 12085 [24].

Compressive stress at 10% deformation (further in the text known as compressive strength) was determined for BcB samples after 6 months of microorganism impact according to EN 826 [25] using a computerised universal testing machine H10KS (Hounsfield, Surrey, UK), which has a maximum loading force of 10 kN, a loading accuracy of ±0.5% and a loading speed accuracy of ±0.05%. Three specimens for each composition with a size of 50 × 50 × 50 mm^3^ were used. Before the test, samples were conditioned for ≥6 h at 23 ± 5 °C.

**Figure 1:**
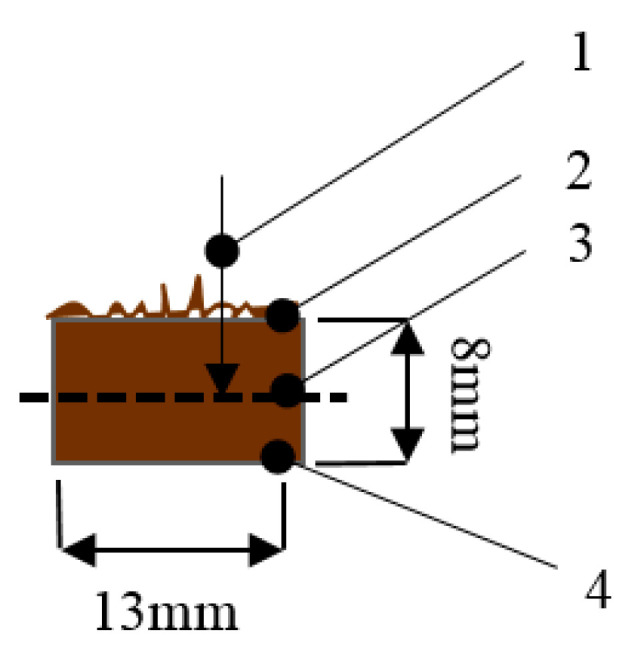
The scheme of the test sample for SEM imaging: 1—direction of investigation; 2—microorganism-affected BcB surface; 3—the middle layer of the BcB sample; 4—the entire BcB sample.

The microstructure of the BcB samples before and after the 6 month growth of microorganisms was investigated with a JSM-7600F Field Emission Scanning Electron Microscope (SEM) (JEOL, Tokyo, Japan). The parameters of the SEM tests were as follows: voltage 4 kV and 10 kV; distance to specimen surface varied from 15 mm to 25 mm; magnification used was 500×, 1000×, 2000× and 3000×. Samples were completely dried in the oven at 60 ± 5 °C temperature for 72 h. Before microstructural analysis, the samples were covered with a thin Au layer using a Quorum Q150R ES instrument (Quorum, Laughton, UK). The growth of the microorganisms in and on BcBs was determined according to the test scheme presented in Figure 1.

Cellulase and amylase activities were initially determined in the Petri dishes with agar. The microorganisms that produce the cellulase enzymes were put on two sets of the 1% carboxymethyl cellulose (CMC) medium (15 g/L agar (Formedium), 10 g/L carboxymethyl cellulose, 1 g/L NaNO_3_, 1.8 g/L, K_2_HPO_4_, 0.9 g/L MgSO_4_·6·H_2_O, 0.5 g/L KCl, 20 g/L (all from Sigma-Aldrich). The microorganisms that produce the amylase enzymes were put onto 1% starch agar medium (SAM, 10 g/L soluble starch (Roquette), 3 g beef extract (Biolab); 12 g/L agar (Formedium)). Then, all sets of Petri dishes with inoculates were incubated for 3–28 days at 22 °C temperature. The cellulase and amylase activities were checked by establishing clear and clearly visible transparent areas around the microorganism colonies showing cellulose hydrolysis. For identification of the cellulase activities, the first set was flooded with Congo red (Sigma-Aldrich), whereas the second one was flooded with Gram’s iodine solution (2 g KI (Roth) and 1 g I_2_ (Sigma-Aldrich) in 300 mL of the distilled water). For detection of the amylase activities, the same KI + I2 solution was used. It took approximately 3 to 5 min to visually see the first results and form a hydrolysed zone.

For the enzymatic activity assays, the enzyme extracts were prepared using 0.25 g ± 0.05 g BcB samples that were affected by microorganisms. For determination of the cellulase activities, 5 mL of 50 mM citrate buffer at pH 4.8 was used, whereas for the amylase activities, 5 mL 50 mM phosphate buffer of pH 6.9 was used. The samples were stirred for one hour on an agitator table at 120 rpm at 22 °C temperature, then centrifuged at 10,000 rpm for 10 min. Further, the sterile filter with cellulose acetate membrane was used to filter the supernatant. Total cellulase activity was determined by the filter paper by adapting the method recommended by the IUPAC [26]. Cellulase activity was measured using 500 μL of enzyme extract with 50 mg filter paper as a substrate and incubation for 2 h at 50 °C temperature. Sugars (released glucose) were measured using 3,5-DNS acid (Alfa Aesar, Haverhill, MA, USA) at 540 nm by the microtiter plate reader or microplate spectrophotometer (EON, Biotek, Winooski, VT, USA). One unit of total cellulase activity was defined as the amount of enzyme that releases 1 μmol of reducing sugar from the filter paper per mL per min. Endoglucanase activity was measured by the carboxymethylcellulose (CMC)/DNS method using the IUPAC recommendations. As the substrate, 500 µL of 2% CMC in the 50 mM citrate buffer at pH 4.8 was used, mixed with the 500 µL of enzyme extract and incubated for 1 h at 50 °C. The released glucose was assayed by the 3,5-DNS acid method at 540 nm by the microtiter plate reader as described above.

To measure the amylase activity, 500 µL of 1.0% (*w*/*v*) starch in a phosphate buffer (pH 6.9) was mixed with 500 µL of enzyme extract and incubated for 15 min at 37 °C. The released maltose was assayed by 3,5-DNS acid at 540 nm by the microtiter plate reader as described above. The calibration curve for detection of the cellulases was performed using the glucose, whereas for detection of amylases, maltose was used.

Experiments to measure the density and compressive stress at 10% deformation of the BcBs and different enzymatic activities were performed in triplicate. To calculate the average values and standard deviations, the Microsoft Excel 2016 and Sigma Plot 12.0 software programs were used.

## 3. Results and Discussion

### 3.1. Identification of Bacterial and Fungal Species

**Figure 2:**
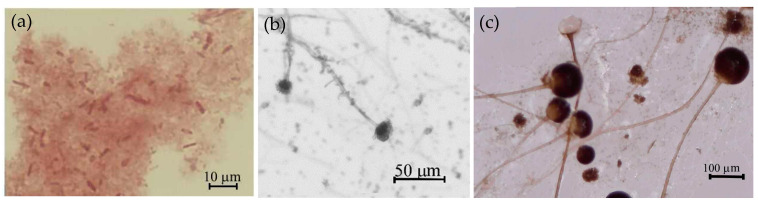
Microbial species seen by optical microscopy (magnification 400× for bacteria and 40× for fungi): (**a**) *P. putida,* (**b**) *A. fumigatus*, (**c**) *Rh. oryzae*.

**Figure 3:**
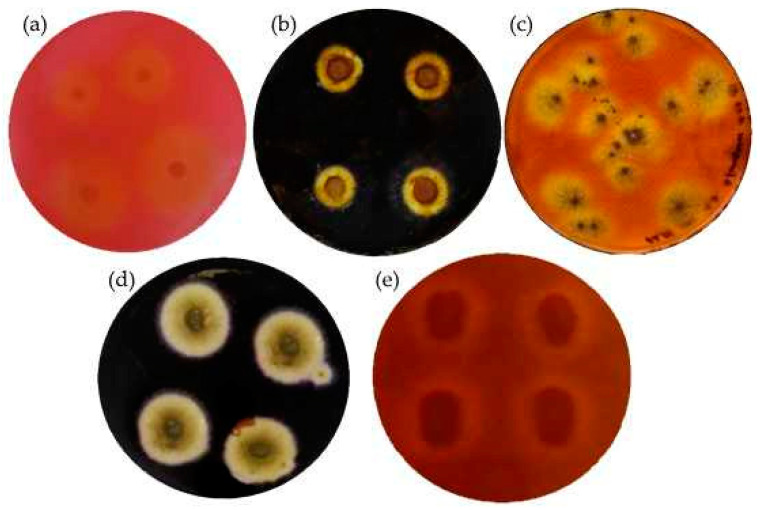
Detection of enzymatic activities from the isolated fungal strain by floating Petri dishes with Congo red for cellulase activity and Gram iodine solution for amylase activity, respectively: (**a**) *Rh. oryzae* after 12 days of growth with Congo red, (**b**) *Rh. oryzae* after 4 days of growth with Gram iodine, (**c**) *A. fumigatus* after 7 days of growth with Congo red, (**d**) *A. fumigatus* after 7 days of growth with Gram iodine, (**e**) *P. putida* after 14 days of growth with Congo red.

In this work, we selected microorganisms (bacterium *P. putida* and fungi *Rh. oryzae*, *A. fumigatus*, Figure 2) that decompose cellulose (Figure 3) by their enzymatic reactions. *P. putida* was selected out of several identified bacteria because it was the only species that possessed cellulose activity. *Rh. oryzae* was selected out of dozen fungi because of literature data that describe it as a good cellulose degrader. Suspension of *P. putida* and *Rh. oryzae* was used to analyse the biodegradability of BcBs, but during the incubation, the unexpected growth of a light-green fungus was also visible. Its identity as *A. fumigatus* was determined by molecular microbiology methods. The results of the sequencing of the 16S rRNR V1–V9 region showed 99.83% similarity to the *P. putida* (Figure 2a); sequencing of the ITS region showed 100% identity to *Rh. oryzae* (Figure 2b) and *A. fumigatus* (Figure 2c).

Since the analysed BcBs contain CS and HS, these composites are an excellent medium for *Rh. oryzae* and *A. fumigatus* to propagate (Figure 2) and produce the enzymes that induce biodegradation. Moreover, previous studies [27,28] have shown that CS and HS are tolerant to inhibitors existing in lignocellulose biomass-based acid hydroxylates and are able to directly utilise cellulose and hemicellulose at slower rates in humid and dry conditions. Previous results [29] have demonstrated the dependency between the microbial community variation and the chemical compositions of hemp fibres and have further shown that fungal colonisation occurs in the first week, whereas very little bacterial growth was observed. The current study determined that the conditions used, i.e., 22 °C temperature and 65% relative air humidity, were not sufficient for *P. putida* bacteria to survive, degrade cellulose and further propagate.

### 3.2. Measurment of Enzymatic Activities

**Figure 4:**
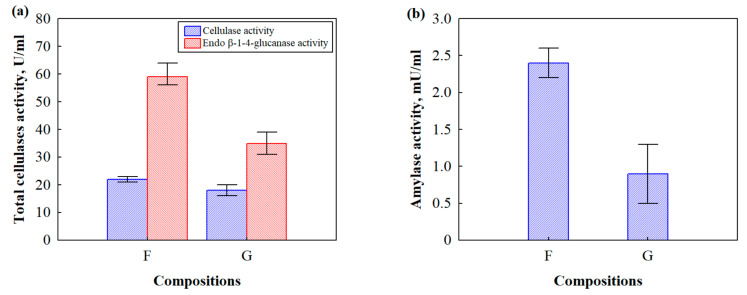
Total enzymatic activities of the microorganisms after growth on BcBs with different flame retardants: (**a**) cellulase activity; (**b**) amylase activity. All measurements were in triplicate; the average values and standard deviations are given.

As BcBs consists of HS, which is a source of cellulose, and CS, which is a source of amylose, it is of great importance to determine the cellulase and amylase activities caused by fungal growth. Therefore, a rapid qualitative method was used to determine the cellulase and amylase activities of microorganisms growing in the Petri dishes. Pure microorganism culture producing cellulase and amylase enzymes were selected using KI or a Congo red test on the CMC. Its degradation was observed by loading Petri dishes with one of the solutions. Cellulase and amylase activities were observed regarding the zone around the microorganism colonies, which showed cellulose hydrolysis. It took 5–10 min to visually see the first results and form a hydrolysed zone, not counting the growth time of the microorganisms [30]. Figure 3 shows the enzymatic activities of *Rh. oryzae* and *A. fumigatus,* and Figure 4 depicts the numerical values of the cellulase, amylase and endo β-1-4-glucanase activities. The cellulase and amylase activities were measured by determining the clear zones surrounding the colonies. It can be clearly seen that *Rh. oryzae* causes higher cellulase activity, whereas the increased amylase activity is mainly determined by *A. fumigatus*. The bacteria *P. putida* showed cellulase but not amylase activities.

After the incubation of BcBs with different flame retardants (F and G compositions) at 22 °C temperature and 65% relative air humidity with microorganism suspension, it was found that F composition was characterised by the highest total cellulase activity produced by both microorganisms. Such activity was by 17% greater compared to that of BcB with G composition. Similar observations for F composition were performed with endo β-1-4-glucanase and amylase activities, which, compared to G composition, were 63% and 52% greater, respectively.

The observed differences in enzymatic activities between BcBs with F composition and G composition can be attributed to the aforementioned pH value of the flame retardant in F composition, which makes for easy access to the enzymatic hydrolysis of CS and HS. According to a previous work [31], the susceptibility of starch to enzyme action occurs due to the low crystallinity of the gelatinized form between CS and HS; therefore, amylase activity in BcBs with F composition is higher (Figure 4b). In addition, cellulose and amorphous regions of the cellulose supramolecular structure also become more accessible for enzymatic action in HS, thus increasing the activities of cellulase and endo β-1-4-glucanase in BcBs with F composition (Figure 4a).

### 3.3. Microstructure of BcBs after Incubation Period

**Figure 5:**
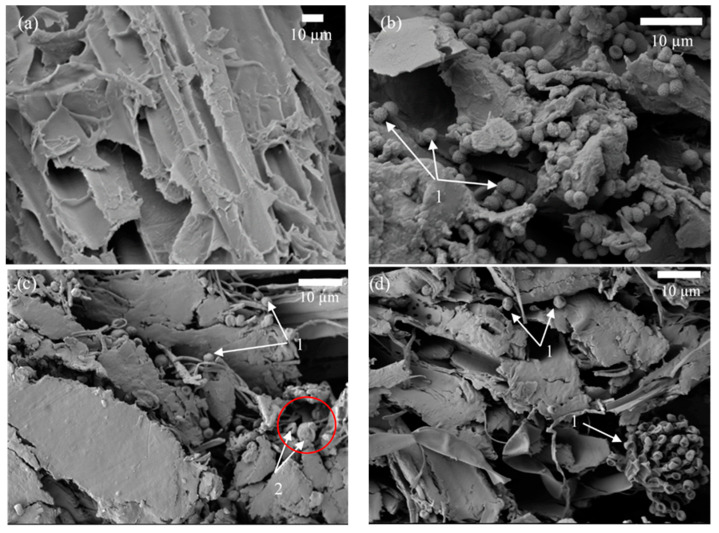
Microstructure of BcB with F composition as determined by SEM: (**a**) before incubation (magnification ×1000); (**b**) 6 months after incubation—1 mm depth of BcB (1—*A. fumigatus*) (magnification ×2000); (**c**) 6 months after incubation—2–2.5 mm depth of the BcB (1—*A. fumigatus*, 2—*Rh. oryzae*) (magnification ×1500); (**d**) 6 months after incubation—the middle of the BcB (1—*A. fumigatus*) (magnification ×1500).

It is well known that HS consist of the polysaccharide cellulose (33–44%) [32,33], the polymer of glucose, which is the main carbon source for microorganisms. In the presence of excess water through the amorphous sites in the cellulose structure [34], water penetrates into the shives. It is likely that microorganisms will also find an amorphous site in the cellulose chain, penetrate and break down the polysaccharide. As a result, the cellulose structure will break down and BcBs will decrease in durability, losing their mechanical performance. Colonisation of the microorganisms on the surface of composites generates different changes in the topography of the surface [35,36]. The most commonly depicted effects include the presence of microorganism colonies and penetration of the mycelium hyphae into the composite resulting in cracking and voids on the surface [37]. Therefore, the examination was performed with a scanning electron microscope to determine whether the microorganisms were growing inside the two different flame retardant-modified BcBs and whether the hemp walls or interface between HS and CS were being destroyed. Therefore, Figure 5 shows the microstructure of a control sample before incubation and after 6 months of incubation in different depths of the BcB sample.

**Figure 6:**
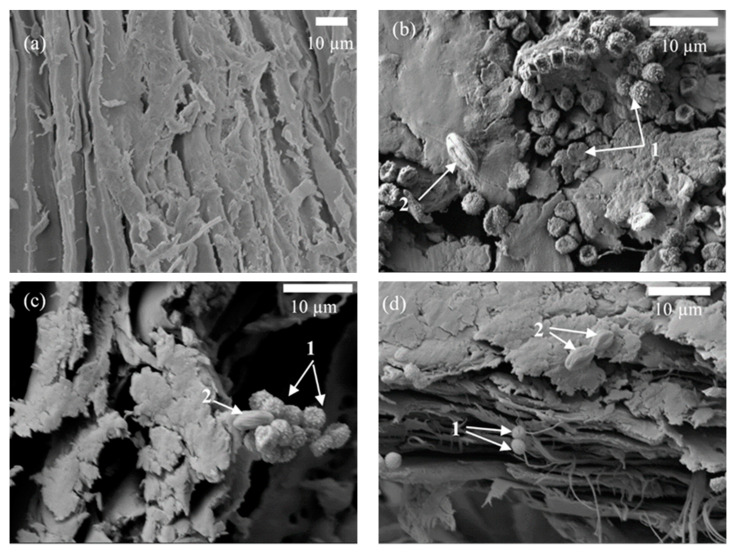
Microstructure of BcB with G composition as determined by SEM: (**a**) before incubation (magnification ×1000); (**b**) 6 months after incubation—1 mm depth of BcB (1—*A. fumigatus*, 2—*Rh. oryzae*) (magnification ×3000); (**c**) 6 months after incubation—2–2.5 mm depth of the BcB (1—*A. fumigatus*, 2—*Rh. oryzae*) (magnification ×3000); (**d**) 6 months after incubation—the middle of the BcB (1—*A. fumigatus*, 2—*Rh. oryzae*) (magnification ×2000).

Figure 5a shows the regular structure of a HS along the direction of stem growth. Basically, HS are characterised by their cellular structure, which is mainly determined by two types of voids—vessels and tracheids [38]. However, the microstructure of BcBs after incubation with microorganisms for 6 months led to the destruction of the structure. It was observed (Figure 5b) that the main cause of destruction at the top layer of the BcB, i.e., 1 mm in depth, is *Aspergillus fumigatus* conidia. The interface between HS and CS was fully destroyed; pores and voids were visible as well. In addition, SEM images of the 2–2.5 mm layer (Figure 5c) showed similar behavior but with an additional impact of *Rh. oryzae* sporangium with spores. The middle layer of the BcB (Figure 5d) showed a severely affected and disintegrated structure, almost no contact zones between the aggregate and the binding material and few conidia and condiophores as well as phialides of *A. fumigatus*. All samples were colonised by the consortia with fungal penetration along the CS layer accelerating the attack on the HS and the entire BcB itself. It is likely that all organic compounds used for the production of BcBs likely served as carbon and energy sources for the growth of fungi.The impact of fungi on polymer biocomposites has been studied previously [39,40], and it was concluded that the cross-section of veneer with large openings could be also a pathway of the fungi. Therefore, vessels and tracheids might induce the decay of BcBs with F composition, thus providing abundant paths for fungal hyphae to penetrate the deeper layers of the product. Further, Figure 6 depicts the microstructure of BcBs with G composition. The original sample has a clean interface between CS and HS (Figure 6a), and no hyphae growth can be observed in the cell cavity.

At 1 mm depth of the BcB (Figure 6b), two types of fungi can be detected. Previously [41], it was shown that fungi attack the surface of the samples via cracks or gaps between the filler and the matrix; therefore, the cracked zones in CS were mostly disseminated by *A. fumigatus* conidia, whereas only few ~6 μm-sized sporangium spores of *Rh. oryzae* were found. The distribution of the fungi colony at 1 mm depth is similar to that of the BcB with F composition, although the destruction of the microstructure is lower than that of the BcB with G composition. Going deeper into the structure, i.e., 2–2.5 mm and then in the middle of the sample (Figure 6c,d), less and less fungi sporangium can be detected, confirming that expandable graphite is a more effective flame retardant with regard to biostability. However, after the six months of incubation, the distribution of scarce hyphae can be observed in the cavities between CS and HS in the middle of the BcB sample with G composition (Figure 6d) and in the 2–2.5 mm depth, as well as in the middle of the BcB sample with F composition (Figure 6c,d). Similar changes in structure were observed for straw fibre/HDPE composite inoculated for 15 days with wood-rotting fungi [42].

### 3.4. Growth of Microorganisms on BcBs

**Figure 7:**
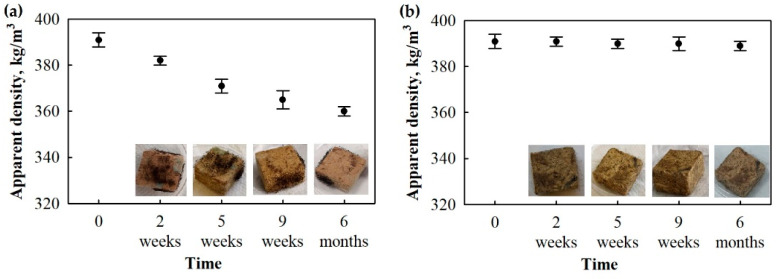
BcB samples after incubation of *P. putida* and *Rh. oryzae* mix at 22 °C temperature and 65% relative air humidity conditions for 6 months: (**a**) F composition, (**b**) G composition. All measurements were performed in triplicates, and the average values and standard deviations are shown.

In order to evaluate the long-term growth of microorganisms on BcBs at 22 °C temperature and 65% relative air humidity, photographs of the microorganism-affected samples were taken after 2, 5 and 9 weeks and 6 months. Figure 7 clearly depicts the difference between the BcB samples with the two different flame retardants, i.e., F and G.

When the suspension of *P. putida* and *Rh. oryzae* was applied on the BcBs, the intense growth of light-green/green and black hyphae of fungi can be observed between the second and fifth week. *Rh. oryzae* appear as black mold, whereas the light-green fungi were identified as *A. fumigatus* by microstructure and molecular microbiology methods.

However, the intensive fungal growth slows down after 9 weeks. In addition, the predominant microorganisms on the surface othe f BcBs are micromycetes. The difference regarding the fungi growth intensity between the F and G compositions may be observed as well. The BcBs with the F composition have an almost fully covered surface with fungi, whereas the BcBs with the G composition remain only slightly affected even after 6 months. It was also noted that within the F composition, as time progresses, there has been some reduction in the apparent densities (Figure 7). However, the parameter remained almost the same for BcBs with the G composition. It can be clearly seen that the degradation of the BcB by fungi happens in the F composition, i.e., the apparent density is reduced by 8% compared to the control BcB. The change in mass after fungal impact was also observed previously [43] for commercially available wood composite panels such as plywood, oriented strand boards, particleboards and medium-density fibre boards. Due to respiration performed by the fungi, cellulosic matter is metabolised and converted into more complex and longer organic molecules, and carbon dioxide and other liquid material that escape from the samples lead to a negative mass change [44]. However, no mass change or reduction in apparent density was noted for the G composition, indicating some barrier effect, which will be further studied.

Surprisingly, no visual bacterial growth was observed in this experiment, even though the *P. putida* strain isolated in this work possessed cellulose activity (Figure 3). In addition, previous works identified *Pseudomonas* sp. as cellulase producers [45,46]. Since we also have not been able to observe *P. putida* in the BcBs directly by SEM, we conclude that the conditions are not favorable for the growth of bacteria. However, we cannot exclude the fact that bacterial cellulases and amylases contribute to the enzymatic activity observed in the BcBs.

It has been determined [47] that humidity levels up to 75% are safe for most building materials if the mixture of raw materials is highly alkaline, whereas acidic pH levels can cause the growth of fungi. BcBs with the F composition contain flame retardant that is slightly acidic, i.e., its pH is approximately 4.5, which might be a cause of the more rapid development of fungi in the F composition samples compared to those with a G composition. Further, the N and P elements that are present in Flovan CGN may stimulate fungal growth. In contraat, the G composition contains expandable graphite that is considered to have sufficient antibacterial properties [48] whose intensity depends on the average size of particles. Previously, it was shown that graphite with an average size of 6.87 μm had approximately 26% antibacterial activity measured by the reduction of cfu during cell viability tests [49]. As the current study uses expandable graphite with 70% of its particles >300 µm, whereas the rest are described as smaller flakes, it can be predicted that the antibacterial activity in BcBs with the G composition is even higher.

### 3.5. Mechanical Performance of BcBs after Incubation with the Microorganisms

**Figure 8:**
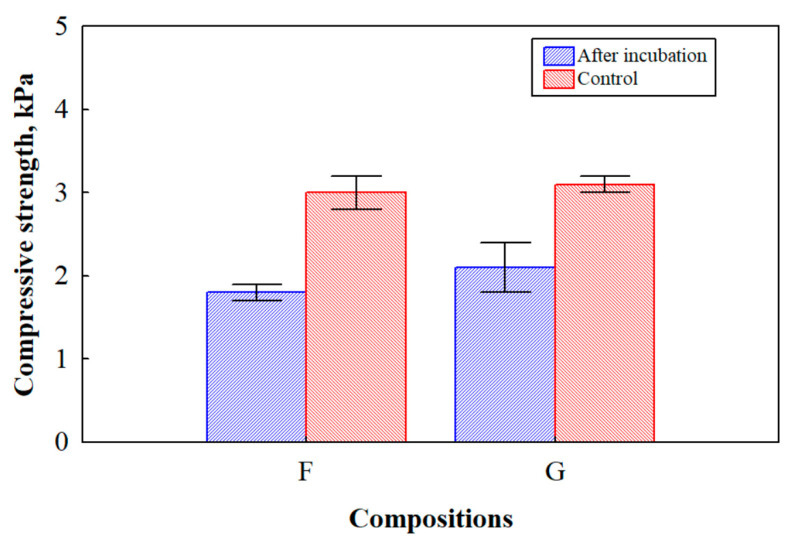
Compressive strength of BcBs before and after incubation for 6 months with mix of *P. putida* and *Rh. oryzae*. Measurements for F and G compositions with or without inoculation (control) with microorganisms are demonstrated. Experiments were performed in triplicate, and the average values and standard deviations are shown.

Since it is very important to obtain sufficient long-term performance of ecological bio-based building materials, it is of concern to evaluate the change in mechanical performance after inoculation with microorganisms and six months incubation. Figure 8 presents the results of the compressive strength of the BcBs with different flame retardants before and after incubation with microorganisms.

Taking into consideration the scattering of the results, the compressive strength of the control BcBs with the F and G compositions was the same, i.e., 3 MPa, and the obtained differences can be neglected. However, the decrease in this parameter after inoculation and six months incubation in the F composition reached 43%, and in G composition 37%, compared to the control BcBs. This can be attributed to the weak interface interaction between the HS and CS caused by the degradation of lignocellulosic components during the fungal attack. This type of degradation resulted in the pores, cavities and other imperfections of samples, consequently causing the reduction in mechanical performance. Similar findings were previously reported [50,51]. Moreover, it is worth mentioning that the difference between the BcBs with F and G compositions after a fungi attack is ~4.5%, leading to the confirmation that expandable graphite has some antimicrobial behaviour.

Interestingly, BcBs with the G composition before inoculation and after six months of incubation had an unchanged apparent density (Figure 7), although compressive strength had been reduced. It can be assumed that lignocellulosic components in these BcBs were not fully degraded nor damaged due to the action of expandable graphite, but the contact zones between CS and HS were loosened, thus deteriorating the mechanical performance of the final products.

## 4. Conclusions

The current study has revealed that microorganisms build up colonies in and on BcBs, especially if the environmental conditions are suitable for the growth of fungi and bacteria. The predominant microorganisms after six months of incubation were found to be *Rh. oryzae* and *A. fumigatus*. Measurements of the enzymatic activities of BcBs with F and G compositions showed that cellulase activity was 22 U/mL and 18 U/mL, and amylase activity was 2.4 U/mL and 0.9 U/mL, respectively, resulting in more severe degradation of the F composition BcBs, with a particularly reduced apparent density (from 390 kg/m^3^ to 360 kg/m^3^) and mechanical performance (from 3.0 MPa to 1.8 MPa) due to the severely affected middle layers and disintegrated structure, whereas the G composition BcBs were more stable based on the cellulases, amylase activities and apparent density. Even though expandable graphite is suitable as an antimicrobial agent for BcB production, its mechanical performance is almost the same as for the F composition BcB. Therefore, neither BcB is suitable as a building material in direct use for structural purposes without additional treatment such as impregnation or lamination, which could reduce the growth of fungi and bacteria and their resulting structural flaws in products.

## Figures and Tables

**Table 1:** Composition of BcBs. HS—hemp shives; CS—corn starch G—expandable graphite; F-Flovan.

Raw Material	Composition		
	Control	G	F
HS, g	300	300	300
CS, g	30	30	30
Boiling water, L	2	2	2
G, wt.%	0	10	0
F, g/L	0	0	30

## Data Availability

Not applicable.
